# Tracheotomy in Pseudo Membranous Croup

**Published:** 1858-07

**Authors:** Thos. F. Rochester


ART. II.—*Tracheotomy in Pseudo-Membranous Croup.* By THOS. F. ROCHESTER, M. D. Report read before the Buffalo Medical Association.

The ætiology and the pathology of croup, although by no means definitely settled, demand a brief notice from your reporter prior to the consideration of the special treatment, which you have assigned to him to lay before you. With this view the following propositions will be assumed as generally recognized by the profession, viz., The distinction between spasmodic and membranous croup is well grounded, still in a few exceptional cases, the discrimination is difficult if not impossible. This fact must not be disregarded, especially in the present investigation, not however, as might be supposed, as presenting an argument against tracheotomy.

Pseudo-membranous croup is especially a disease of infancy and early childhood, it rarely attacks nursing children, its subjects are in the vast majority of instances from one to five years of age, the common notion that it is mostly confined to the strong and vigorous is incorrect, as delicate children, especially if strumous, are frequently its victims; if there is a class more liable to its visitation than another, it is composed of those of lymphatic temperament with a tendency to obesity. Temperature, season of the year and locality, exert a marked influence upon its prevalence and its character. It is seen most frequently in spring and autumn, it is apparently often developed by exposure to high and cold winds, it exists equally, numerically, in low and moist and in dry and elevated situations, presenting however corresponding differences in grade. This is well illustrated in the cities of New York and Brooklyn, which are separated only by the Hudson river. In that portion of the latter city known as the Heights, croup is much more violent, severe and rapid in its course than in New York. The comparison holds true, on a larger scale, in observing the affection in the mountainous regions of New England and the extended plains of the Western States.

Croup is both indiopathic and epidemic, it is both primary and secondary—it is associated with or succeeds, influenza, catarrh, pharyngitis, rubeola, scarlatina, varicella, variola, and other diseases. It is occasionally sudden in its onset and rapidly fatal. Its course is usually however insidious, presenting catarrhal symptoms for several days before it is fully declared. It is essentially paroxysmal in its phenomena, presenting marked nocturnal exacerbation. It is attended with hoarse cough and voice, the former gradually changing to harsh, dry, metallic or ringing, and the latter to husky and whispering. Both may become entirely extinct. In two fatal cases, attended by the writer, voice was nearly extinct from the commencement of the attack and cough was slight and infrequent. Similar instances have also been noted by others. Respiration is usually accelerated and embarrassed, often to an extreme degree, but with intervals of comparative ease. Febrile movement, varying greatly in intensity is the rule, but even to this there are exceptions.

In view of the foregoing propositions, which arc assumed as recognized by the profession, it is difficult to define the precise pathology of the disease in question. Is it simple laryngo-tracheitis? The Viennese school regard it as a local manifestation of a general disorder or diathesis, and Rokitansky in his list of "Dyscrases" subdivides the "croupous crasis" into three forms, the minor divisions representing certain quantitative or qualitative changes in the fibrine element of the blood. This view presents at least a plausible explanation of the manifestation of the affection under various and diverse exciting agencies. From the distinctive designation of the disease (pseudo-membranous) it would appear superfluous to inquire whether it is always attended with exudation of false membrane, but reliable statistics demonstrate that it is *entirely wanting* in a small proportion of cases, being neither detected during life nor on post-mortem examination. A knowledge of this circumstance induced Blood to classify the affection as follows: "*Myxogene*, when mere mucus is expectorated; *Pyogene*, where pus is effused; and *Meninyogene*, when the membrane is formed." The fact of the non-formation of the membrane, in a certain proportion of fatal cases, appears to the writer of sufficient importance to warrant him in citing the authorities from which it is derived, the more so as it illustrates one cause of death which tracheotomy would, perhaps avert, viz., long continued or frequently repeated spasm of the glottis.

"Practically, therefore, as well as pathologically, we cannot say with Bouchut, 'without a false membrane, croup does not exist.'" (Peaslee Amer. Med. Monthly, January, 1854.)

"Where the disease terminates quickly in death, no well formed false membrane is seen, but only mucus in the trachea more or less thick, and redness about the glottis." (Prof. Alexander H. Stevens, Buff. Med. Jour. Vol. 2d, p. 613.)

"Six of the fatal cases were examined after death; in one there was no false membrane *anywhere*, but intense redness of the larynx trachea and bronchi, with an uneven granular appearance of the larynx and ulceration about the epiglottis." (West on Diseases of Infancy and Childhood, p. 218. Eng. Ed. 1848.)

In addition to the above, Dr. A. W. Nichols informs the writer, "that in 1856 he made a post-mortem examination of a child, under two years of age, who died of croup in this city, after an illness of twenty four hours. It had never expectorated any false membrane, and none was discovered —there was redness and tumefaction of the larnyxo-tracheal mucous membrane, and a plug of inspissated mucus just above the bifurcation."

"There are a few cases in which this adventitious membrane is not formed at all." (Watson, Lecture 46.) "This membrane or layer of lymph does not always present itself in the worst cases." (Dickson's Elements, p. 592.) It is certain however that in the vast majority of cases, a false membrane is developed, varying however very much in extent, character and thickness. It is often limited to the larynx, but it is likewise often continued to the minutest bronchial tubes. It is frequently firm and tenacious, containing a large proportion of fibrin, while it is as often of a muco-puruloid character, representing a state of partial solution or decomposition; it is not organizable; it is often reproduced; it varies from extreme tenuity, to a line and a half or two lines in thickness; it is grey or dirty yellow in color, the subjacent tissue is always red and tumified, but rarely eroded. *The membrane or membranes are rarely so thick as to cause death by occlusion of air.*— The writer has conducted six examinations of fatal cases, he has witnessed at least as many more; in none was there complete obstruction, and in most of them it was comparatively slight. Dr. S. S. Bemiss, in an excellent "Essay on Croup," published in the Louisville Review, says: "It is worthy of remark, that in a majority of dissections of patients dying from croup, the mechanical occlusion of the glottis, by either the membrane or its subjacent swelling, is not so complete as to account for death." This is also intimated by West. Marshall Hall refers the fatal termination in many instances to œdema-glottidis. In one of the cases observed by your reporter, the membrane was small and attached only at its upper terminus—it was, as it were, a strip of narrow tape dropping from the chink of the glottis into the trachea. The idea at once suggested itself, that this could not seriously interrupt the inspiratory act, but that it would greatly impede the process of expiration, by its valvular action.

The preceding assertions being well established, is it not evident that in addition to membranous obstruction, spasmodic occlusion of the glottis must also be taken largely into account, admitting at the same time, that the presence of the membrane is the principal cause of the spasmodic closure?

It is next important to consider, the primary seat of the exudation, and the limits to which it is usually confined. It is the general impression that the pharynx rather than the larynx is first invaded, that the characteristic patches may be detected upon the tonsils, uvula or fauces for some hours or days even before they attain the air passages proper. Trousseau is strenuous upon this point, scarcely admitting an exception to the rule; he describes very aptly the manner of extension, comparing it to the spreading of a drop of oil upon paper. Unhappily however for the purpose of early and certain diagnosis, the exudation frequently commences in the larynx itself. West says: "The fauces do not present any invariable alteration in cases of croup." He further states "that of ten idiopathic cases under his own observation, two only presented a scanty formation of false membrane upon the velum and tonsils." Dr. J. F. Meigs had nineteen cases under his own care, "of these, the croup followed membranous angina in eleven, in five the disease *began* in the larynx and thence extended to the fauces, and in three there was no deposit on the throat at any time." Further evidence on this point is judged unnecessary. With reference to the seat as well as to the extent of the membrane, statistics are abundant. In 54 antopsies M. Betonneau found the false membrane terminating in different points: in the trachea in 31, penetrating the large bronchia in 16, reaching the terminal bronchia in 7. In a table of 171 cases, collected by M. Guersent: in 78 the membrane terminated in the trachea; in 42 it invaded the bronchia; in 30 the condition of the bronchia was not mentioned; and in 21 there were no false membranes. (Meigs.) The above statistics are sufficient to show that in a considerable majority the bronchia are not implicated.

*Complications.* M. M. Barthez and Rilliet (*Maladies des Enfants*) found pneumonia in five-sixths of their fatal cases. Bronchitis is very frequently present. Exhaustion from defibrination of the blood is mentioned by Rokitansky, and W. B. Richardson (London Lancet, 1854) speaks of ante-mortem fibrinous heart clots reduced by repeated paroxysms of partial asphyxia.

*Mortality.* This is always large, and fearfully so in certain districts and under certain epidemic influences, amounting to a totality of all attacked in one instance mentioned by M. Ferrand, where in sixty cases there were sixty deaths. (*Dict. de Méd. Vol. IX. p.* 364, 399.) It is difficult to arrive at the average mortality, both on account of the conflicting statements of various writers, and on account of the influence of treatment in averting, or possibly, too often, in producing a fatal issue. It is probably rather below than above the mark, however, to assume that one in three die. M. Valleix found in fifty-four cases of undoubted croup but seventeen recoveries. (*Guide du Médecin, Vol. I. p.* 200.) Dr. Bard of New York lost seven out of sixteen. Dr. J. F. Meigs, ten out of twenty-two. West, fifteen out of twenty-two. Ware, ten out of fourteen. Clark, two out of five. Horace Green, three out of nine. It does not come within the scope of this paper to discuss the merits of the various forms of treatment that have been directed against this terrible scourge, it is with tracheotomy alone that our attention must be occupied for the remainder of this article. The most decided advocates of this measure are found among the French practitioners. It was first instituted, so to speak, in France, A. D. 1822, by M. Brettonneau of Tours, who met with sufficient success to induce many others to follow his example. The most earnest, as well as by far the most experienced, of the supporters of tracheotomy in membranous croup is M. Trousseau of Paris. It is somewhat to be feared that from the circumstances of his position he has been placed in the attitude of a partisan or champion, rather than that of a candid and impartial observer. From the reports made by himself and from information pertaining thereto gathered from various sources, there appears to be a discrepancy, or at least an obscurity, as to the actual measure of success. Thus, M. Trousseau made one hundred and thirty-five operations from 1830 to 1850, of which but one in four were successful. (*See L'Abeille Médical*, June, 1852.) The statistics of the *Hospital des Enfants*, from 1850 to 1855, show 216 operations and 47 recoveries, less than one in four. This is claimed, however, and justly, as a good result, considering the class of patients and their unfavorable hygienic position, and it is assumed by the editor of the *Annuaire des Science Médicales*, 1856, that recovery would succeed half of M. Trousseau's operations in private practice, and substantiating this view, we find in the *Archives Général de Médecine*, 1855, this statement made by M. Trousseau, " that from 1851 to 1854, he had 15 operations and 7 recoveries, while in 1854 he had the unparalleled good fortune to save 7 out of 9 by this method." He attributes his increased recent success to improvements in the canula employed and to special care in the after treatment. With reference to the operation itself and the cases in which it is proper, Dr. Walter Atlee of Philadelphia has furnished some excellent notes from a clinical lecture delivered by M. Trousseau in 1852; from these the writer will venture to quote largely, as follows:

" *Indications for this Operation.* When the affection has commenced in the pharynx and has extended slowly, in such cases the recovery is so rare without tracheotomy that you must operate. You must not wait for the last moment of asphyxia. When the cough is rare, generally extinguished, when the respiration is hissing, when there are orthopnœa and the paroxysms of suffocation, then you must operate. * * * * I have not hesitated to perform the operation upon children dead, that is to say, no longer breathing, and they got well. This extreme degree of asphyxia is an inconvenience, but it is not a formal contra-indication. When the disease has progressed slowly hope much; when suddenly do not hope. When there are false membranes on blistered surfaces, the nose, &c., it rarely if ever suceeeds. As to the age, it is from three to five years that you count the greatest number of cases. * * * *Under two years of age* the operation is quite useless; they die almost always of convulsions.

*For the operation.* You must have a matress on a hard and flat table, no pillow, but a block of wood, under the neck. The instruments you require are a common bistoury, a blunt, pointed one, some hooks, a dilator and some canulas, the canula to be double and its diameter as great as possible consistently with the age of the patient; a proper number of aids; and you must have a *tallow* candle—never use one of wax—to perform an operation. The block must be placed under the first dorsal vertebra. The child has always the time to be operated upon *slowly;* trace a line with a burnt cork; then open the skin, perfectly at your ease. You cut carefully between the muscles, until at last you perceive the thyroid gland lying across the trachea; you cut the gland and a small jet of arterial blood spouts; but do not trouble yourself with that. At last you see the rings of the trachea at the bottom of the wound. Now suppose a case in which the veins trouble you; if you cannot avoid them cut them. The jet is violent but soon ceases; cut gently and use the sponge after each stroke. Ordinarily you come upon the first ring or the cricoid cartilage. You lay bare three rings, with the bistoury; you make a hole the size of a pin's head; then you introduce the blunt, pointed bistoury, and finish. You make use of the dilator, and at the end of a minute you introduce the canula.

*Dangers of the operation.* What are the dangers that frighten the young physician and that ought to do so? It is rather the operation of the physician than of the surgeon, one of those little operations that a physician ought to perform. You have time enough, I repeat, you must work slowly. Of all the accidents, the gravest is the cutting of the carotid. It happens occasionally that the carotid takes an anomolous course, but if you perform the operation as you should perform it, there is no danger even then. Twice in my life I have encountered anomolies and I have drawn aside the vessel with a hook. The venous hæmorrhages, are they dangerous? They exhaust the child and they make the physician lose his presence of mind. When you cut veins do not tie them, it is useless. Put your finger on the bottom part of the wound, sponge and *wait.* Hold the child up; quiet him a little.

*You have time enough.* You may attack the trachea too violently, it is not as large as the finger and flexible. Be gentle, cut by very slight strokes, and then use the blunt, pointed bistoury; with it you can cut nothing else. If the child has an attack of syncope, wait. When the trachea is opened the blood enters. This is by no means a great inconvenience. Open the trachea with the dilator; the child breathes well and the flow of blood ceases. After the operation you often see an attack of syncope, but it is nothing. Immediately after the canula is introduced it becomes a source of irritation, but that passes off. * * * * If at the end of a quarter of an hour there is a respiration analogous to the sound of a saw, sawing stone—the serratic respiration—death is certain. If the pulse at the end of twenty-four hours becomes extremely frequent, the children die. When the child is seized with convulsions he dies. After twenty-four hours the *wound* generally swells a great deal, it is a favorable sign; if it does not swell the children die. There is a complication, an enormous swelling of the *neck*. You must ward it off by cauterising the edges of the wound at the end of twelve hours. You must repeat the cauterisation three or four times. If this enormous swelling takes place the children die invariably."

(M. Trousseau in his most recent cases has protected the wound by placing a piece of oiled or waxed silk entirely over the wound; the canula passing through an orifice left in the silk for that purpose, this prevents any chafing of the cut edges of the skin, by the retaining tapes, or by the flanges of the canula.) The after treatment, to which M. Trousseau attaches immense importance, consists in wrapping a coarsely netted woolen cravat around the throat; the air is warmed in passing through this, and neither irritates the wound or the trachea as cold air does. "The cravat should extend over the chin and the upper part of the chest, thus the air entering the lungs will be warm and moist. * * * The great number of successful cases in three years ought to be attributed to two things: the cauterisation and the cravat." Sustaining diet, tonics, if there is any cachexia, a temperature of 70° to 75° Fahr. and good ventilation constitute the remainder of the treatment. M. Trousseau has abandoned topical applications through the artificial opening. He has employed them a great deal and has concluded that they are more frequently injurious than beneficial. M. Trousseau is very precise in his directions with reference to alimentation. The children often refuse food; he does not hesitate to employ intimidation: he first directs milk, eggs, ice cream and broths. If fluids pass into the larynx, he gives very thick soup, vermicelli, hard boiled eggs, large pieces of tender beef, et. cet. M. Trousseau is warmly supported by large numbers of his medical *confrères*. M. Valleix (Vol. 1, p. 200) says:

"At this time, there have been so many successes that no one thinks of forbidding the operation except by figures," and he further adds (loc. cit.) "that with M. Bricheteau he finds that tracheotomy has succeeded in one-third of the cases in which it has been practised, but that there is a consideration of great force which gives increased importance to tracheotomy, to wit, that in the immense majority of cases, the operation has been made under most unfavorable circumstances, when all other treatment had proved ineffectual, and when the gravity of the symptoms and the imminence of asphyxia indicated approaching death. Who does not see that a single cure, under such circumstances, has much more weight than many others obtained in cases where from the outset it has been possible to have recourse to all the resources of art?"

Taking M. Trousseau's own account of the operation itself, it is not without difficulty and danger, although he himself speaks of it so lightly. Upon this point M. Guersant is subjoined, and his experience is only second to that of Trousseau.

"*M. Guersant*, For some years past tracheotomy has been in favor. For my part, I have in the last two years obtained the following results at the *Hospital des Enfants:*

| | | |
|---|---|---|
| In 1850, | 20 operations, | 7 cures. |
| In 1851, | 30 " | 13 " |
| In 1852, | 59 " | 16 " |

"Although the success the last year has been less gratifying, we are strongly induced to perform this operation. It is however a more grave operation than we might suppose; in the first place, because no bloody operation is a slight one, and further, because tracheotomy brings with it numerous difficulties and dangers.

"A child is presented to us having the croup; it exhibits no false membranes which can be detected, notwithstanding it is threatened with asphyxia. We open the trachea; but either from slowness upon our part, or from want of strength upon the part of the little subject, there is inspiration of blood; respiration continues embarrassed for four or five hours, and death is the consequence, although we may have introduced the canula, and effected inspiration through it, this has availed nothing, the blood having fallen into the bronchial tubes. The autopsia reveals to us the existence of false membranes upon the epiglottis, and a clot in the bronchial tubes, and it is probable thut without this accident the child would have been cured.

"Tracheotomy offers difficulties of more than one kind, especially if we operate upon a dark day, in a badly lighted apartment (the light should come from above,) if the child is very young and we are unable to make a sufficiently long incision. In this last case in fact, we grope our way in the dark, we lose time, hæmorrhage weakens the little patient, and that which is still worse, he perishes by asphyxia when the blood falls into the bronchia. This is an accident which has happened to us, an accident that we do not fear to avow, for we regard it as very important that operators publish their faliures as well as their success.

"Tracheotomy is often followed by death; this unfortunate result is attributed to the delay of the operation, to the feebleness of the child, and also to other causes; but I think the inspiration of blood, and the loss of blood, are the principal and the most frequent causes. It is this accident (the inhalation of blood) that I wished to point out in making this communication to you."

To this opinion of M. Guersant, M. Bayer, M. Velpeau and other eminent Parisian surgeons entirely subscribed. To return again however to M. Trousseau, for this appears to be the proper place to give his exact position. He has until quite recently advocated making the operation very early, as soon in fact as there was positive evidence of the formation of the membrane, and in those cases, purely laryngo-tracheal, where certainty was out of the question, not to hesitate or delay, but to take the totality of the symptoms and to act upon the presumption afforded by them. Judging from his latest contribution, entitled "Tracheotomy in the extreme period of Croup," he has modified his views, for he says:

"The idea that the operation rarely succeeds at this time is erroneous. * * * If the diptheritic infection has profoundly attacked the constitution, if the skin and especially the nares are occupied by the specific inflammation, if a frequent pulse, delirium and prostration, show the system to be deeply poisoned, and if the danger is rather from the general condition, than from the local lesion of the larynx and trachea, *the operation ought never to be attempted*, for it is invariably followed by death; but if the local lesion constitutes the principal danger of the disease, it matters not what degree the asphyxia may have reached, and had the child but a few moments to live, the operation succeeds almost as well as if it had been performed three or four hours sooner."

In the Amer. Med. Monthly, Nov., 1857, translated from the *Union Médicale* of Aug. 27, 1857, there is an interesting discussion following the report of M. M. Trousseau and Blache, upon a paper on topical medication by M. Loiseau.

The following extracts are presented as of special interest and pertinent to this report.

"*M. Trousseau.* The numerous cures obtained by means of tracheotomy have latterly removed the kind of interdiction which was held over this operation. Still, it is not yet adopted by all, and it is practised by very few of our profession. Most decline to operate either because they consider the operation in itself too difficult, or because its utility is not sufficiently demonstrated. * * * * *En résume*, the operation of catheterism of the larynx, which is the principal point in the memoir of M. Loiseau, is considered as a very good means for taking the place of tracheotomy, and at all events to be tried before that operation."

"*M. Depaul.* M. Loiseau's method is nothing new, and is not equal in value to tracheotomy.

"*M. Trousseau.* I believe with M. Depaul that the operation of tracheotomy is preferable. All are not of this opinion. In England, this operation is ignored by both surgeons and physicians; at London, at Liverpool, at Glasgow, the operation of tracheotomy is never performed. * * *"

"*M. Velpeau.* * * * Thanks to this memoir we know that croup can be cured without tracheotomy; that is a great deal."

In the opinion of the writer, the following remarks, made in the same discussion, merit very serious consideration.

"*M. Piorry.* The operations upon the larynx for diseases which are most frequently only secondary, are perhaps too much esteemed. In these cases the operations only shorten life, for they are useless in curing the primitive lesion. * * * The operation of M. Loiseau, which at least is exempt from the *dangers of tracheotomy*, is preferable to this last.

In commenting upon the foregoing discussion, Jules Guerin, the editor of the *Gazette Médicale de Paris*, writes:

"But we repeat we have a moderate (i. e. small) confidence in the employment of a mechanical expedient against a suffocation which is not mechanical but morbid, the cause of which persists after as before tracheotomy. M. Loiseau's method is at the same time a mechanical and a curative means. * * * It is a powerful, energetic means, the only one which up to this time has really succeeded. * * * Laryngeal cautensation is then in this respect much superior to tracheotomy."

M. Loiseau's method, so called, consists in protecting the first phalanx of the left forefinger with a metallic shield; the epiglottis is raised and the arytenoid cartilages are *held* apart by the tip of the guarded finger, and with this as a director, the sponge is carried into the laryngeal cavity. It is,an ingenious application of Dr. Green's treatment, and as such deserving of commendation, but *the treatment* was instituted by Horace Green, M. D., of New York, and M. Loiseau and his *confrères*, in virtually ignoring Green by the adoption of a special and selfish nomenclature, display either an unpardonable ignorance, or are guilty of a plagiarism unworthy of scientific men. That they are not ignorant, is self evident from the foregoing report itself, for in it, M. Trousseau refers to Dr. Green's mode of exposing the epiglottis and of passing the sponge probang into the larynx. It is not positively stated, but it is left to be inferred from the context, that Dr. Green was not and had not been in the habit of treating membranous croup after what is with so much effrontery termed "the method of Loiseau," and on which Green had published a memoir in Sept, 1848, or almost ten years before. Whether Dr. Green's treatment has proved or will prove as successful as has been sanguinely anticipated, is, to say the least, doubtful, but the honor and the whole honor of intra-laryngeal applications is justly his due, and the desire to state the simple truth must be the apology for this digression. The operation of tracheotomy, for the relief of membranous croup, is rarely resorted to in England, and nearly as infrequently in the United States. The reason of this abstinence rests mainly upon the well grounded conviction of the utility of general and topical treatment, (cauterisation,) derived from numerous recoveries of persons suffering from all forms, grades and stages of the affection; which recoveries in the vast majority of instances may fairly and solely be attributed to the measures employed; for although isolated cases of spontaneous recovery are occasionally presented to the observer, there is probably no other "self limited disease" which would so frequently find its terminus in death; and yet it is doubtful if the average of mortality from this cause is greater in the last mentioned countries, where more reliance is placed upon general treatment, than in France, where it is abandoned much earlier. Again, as compared with France, tracheotomy is in disfavor on account of its unfortunate results; but these disastrous terminations should not of necessity be ascribed to tracheotomy. When the time and the circumstances under which the operation has been made by the English and American surgeons are considered, it will be found, generally, that the patient was moribund, and that a respite rather than a rescue from immediate death, was the inevitable consequence. In the few, the very few instances in which the trachea has been opened judiciously, when all other measures had been sensibly employed, but not madly or despairingly protracted, it will be seen that equal success has attended the careful and timely operator wherever he may have been, and that there is no just ground for the opinion that better results are obtained in Paris, because the disease itself is "*different there*" from the same generic affection in London and New York. That M. Trousseau asserts too much when he states that "tracheotomy is ignored in Great Britain," it is only necessary to cite the able article of Mr. Lawrence in the London Lancet of Dec., 1855, in which he has collated 216 operations, of which 47 were successful, or about one in four and a half. There is no doubt however that the opinions and the experience of the first medical men in the United Kingdom have been and are against the operation. Among these may be mentioned, Williams, Ryland, Cheyne, Copeland, Erichsen and Porter, the last of whom says, "I have known and heard of it often, but never understood that it produced a recovery." The last quotation is taken from Dr. J. F. Meigs, on "Diseases of Children," and a little further on the same writer says:

"In a paper on tracheotomy in croup by Mr. Henry Smith, (London Med. Times and Gazette, Jan. 26, 1856,) that writer states that he read a paper on this subject before the London Medical Society in the spring of 1853, when a very large number of the Fellows were present, and earnestly and warmly discussed the question, as to whether the operation was justifiable or otherwise. Although my own experience of it had extended over several cases, all of which were unsuccessful, the opinion arrived at by the majority of the speakers was, that the operation was justifiable."

He goes on to say that the result of this discussion induced him to give further trial to the operation in several succeeding instances to which he was called:

"The result however of the proceeding in cases of pure croup has been disheartening, not one recovery having taken place; and doubts have arisen in my own mind of late, as to whether the surgeon is justified in performing a severe and dangerous operation, when the results are so unfortunate;" he concludes however by saying: "If it be clearly ascertained that the lungs are sound, that the morbid appearances are fully developed about the fauces and death from obstructed respiration be imminent, I would not hesitate to resort to tracheotomy, and even if one life out of ten, or one out of twenty be saved, the surgeon will have his reward."

Dr. Charles West, (Diseases of Infancy and Childhood, 2d London Ed. p. 253,) while he does not advocate the measure, does not unite "in the unqualified condemnation of the operation," and the writer is happy to find that his own views with reference to the danger of spasm of the glottis, have been previously entertained by this eminently thoughtful and practical observer, who states the case clearly and fully in the following sentences:

"Among the arguments against the operation, too much importance appears to me to have been attached to the statement of Dr. Cheyne, that three-eighths of the aperture of the larynx have been found free in fatal cases of croup, and that, consequently, there must have existed during life room enough for the entrance of air. I apprehend that bronchotomy is not performed, on the gross mechanical principle, of removing from the windpipe a quantity of matter which prevents the entrance of air into the lungs, but that it is done rather to obviate the dangers of that spasm of the glottis, which the inflammation occasions, and which will not cease, until either the inflammation is subdued, or the spasm relaxes with the approach of death. Even the narrow opening made into the trachea, often much narrower than the aperture of the larynx, though diminished by swelling or encroached on by false membrane, suffices to admit all the air which the patient needs, and for a time at least the dyspnœa is relieved. But," he continues, "the delicate mucous membrane of the bronchi, in the vast majority of cases, is exposed to the cold air of a ward of a hospital, or of a large chamber; bronchitis is thus excited or aggravated and this secondary affection proves almost invariably fatal."

He then suggests that if the immediate contact of cold air could be guarded against, the mortality from bronchitis would be much diminished. This it will be remembered is now avoided by the muffler of Trousseau.

Let us now turn to our own records and statistics. The number of reported cases is but small, undoubtedly much less than the number of operations that have been made. Sufficient data are afforded, however, for safe deductions. Dr. John F. Meigs (Diseases of Children, 3d Ed. 1858, p. 109) has collected from various sources the results of thirty operations; of these, nine were successful—not quite one in three. Four of these were under his immediate care; two recovered; of them he speaks in this wise: "In the four cases that occurred in my own and my father's practice, I have no hesitation in believing that death would have been an almost inevitable result in all, but for the operation, whereas by that two were saved." Dr. M. advises the operation, "when all medical means have been faithfully tried and have proved powerless." In addition to the above thirty, the writer has collected eleven cases, most of them of very recent date, and it is believed that none of them were comprised in Dr. Meigs' list. Eight of the eleven died, giving an increased ratio of mortality over those reported by that gentleman. Your reporter has found several cases of reported membranous croup successfully treated by tracheotomy, but they all occurred in persons at or about the age of puberty, and nothing is cited to distinguish them from simple laryngitis or œdema glottidis, and to this class he thinks should be referred one of the successful cases mentioned by Dr. Meigs, although the patient was but eleven years of age. The operation was made by Dr. Gordon Buck, Jr., of New York. The lad was ill with scarlet fever in May, 1849; he was profusely salivated with mercury, which caused destructive ulceration of a large portion of the contents of the buccal cavity, and produced abscesses in different portions of the body. While in this condition and five weeks after the attack of scarlatina he had "symptoms of croup." These were relieved, but returned in a few days with violence and steadily progressed, up to July 8th, when he was first seen by Dr. Buck. The symptoms then presented were certainly those of an advanced stage of croup. Tracheotomy was performed to the instant relief of the sufferer; he had however a very tardy convalesence, and eighteen months after the operation was unable to dispense with the tube, as with it closed he could only take eight or ten inspirations, the last with considerable effort. Was not this in all probability an instance of laryngitis with ulceration and œdma? It is unfortunate that cases like the above are confounded with those of pure membranous croup, as they perhaps unfairly angment the number of successful operations. A review of the whole number of cases in which tracheotomy has been made, discloses, with scarcely an exception, an immediate and marked improvement in all the patients, continuing from a few hours to four or five days. In those who recover, convalescence is very slow, and usually from three to six months elapse, and after a much longer period, before the voice is entirely restored.

Of the effect of tracheotomy upon "patients already dead," as Trousseau says, a striking instance is reported by Dr. C. N. Andrews of Rockford, to the State Medical Society of Illinois.

"A little girl five years of age presented all the signs of membranous croup. It was decided to operate, but before the preparation could be made, the child was *in articulo mortis*. When the incision was made into the trachea she had ceased to breathe, there was no pulsation of the heart, and she was apparently dead. Dr. Andrews inflated the lungs with a female catheter. At the end of twelve minutes the pulsation of the heart returned. In thirty minutes there was an effort at voluntary respiration, which soon became established. The inflation was then suspended, but had to be renewed at intervals. The patient revived, ate and drank and played with her toys. In this way she lived for about twenty-three hours. The canula occasionally became obstructed by false membrane, and the inflation had to be repeated at intervals. She finally became comatose and died."

This certainly was a most remarkable resuscitation, and it is not impossible, had Trousseau's muffler been applied, but that recovery might have ensued. It is not stated whether an autopsy was made. From the long asphyxia a heart clot may have been formed. The writer will now venture to submit at length the following case, reported by C. E. Buckingham, M. D., of Boston, in the Boston Medical and Surgical Journal of Feb. 11th, 1858, as representing an instance of severe and *pure* membranous croup, in which medication was fairly and judiciously employed, and, that failing was succeeded by a prompt, timely and successful operation.

"Alexander D., two years and seven months old, began to have cough and harsh respiration on the 1st day of January, 1858. I first saw him at 5, A. M., on the 4th. During the morning of the third he had taken several drachms of hive syrup without experiencing any effect. During the night of the 3d he had taken, in divided doses, ninety grains of powdered ipecac and four grains of calomel, but without any attempt to vomit. When I first saw him his voice was husky, the respiration difficult, more particularly the expiration, but there was no cough. The muscles of the neck and chest were in very strong action. There was considerable lividity of the surface. Auscultation gave a peculiar loud hissing sound all over the chest. He was constantly changing his position. The tonsils were quite red, and *free* from lymph. The back of the pharynx was completely lined with lymph, which also covered the epiglottis. The whole surface, so far as possible, was at once thoroughly cauterised with solid nitrate of silver, which produced retching and vomiting, during which false membrane and much mucus were rejected. At 10, A. M., he seemed better. There was very much less difficulty of breathing, and he was more quiet. I sponged out his pharynx with oil at this time, and again in the afternoon, removing a considerable quantity of membranous matter and mucus. Toward night, the respiration became very much labored, and the pulse very rapid and occasionally intermitting. For twelve hours he had taken five grains of chlorate of potassa and one grain of iodide of potassium, hourly. The medicine was stopped at 7, P. M. Except when examining or operating npon his pharynx, he did not cough at all. The difficulty of respiration increasing, the pulse being feeble and more decidedly intermitting, the lividity growing more marked; at 11, P. M., with the assistance of Dr. J. M. Phipps, I opened the trachea, just below the cricoid cartilage, beginning the incision below and cutting four rings of the trachea from below upward. The trachea tube was too large for insertion, and the inner tube only was introduced, and secured with tapes about his neck. The rolling and rising of the trachea during his attempts to breathe, rendered it impossible to enter the knife, until a tenaculum was thrust into it, with which Dr. Phipps held it securely. The instant the trachea was perforated, air rushed in with the same sound that one hears at a *post-mortem* examination, on perforating the pleural cavity. Previous to introducing the trachea tube some shreds of lymph were drawn out from *below* the incision. Not more than two or three drachms of blood were lost. Ether was administered before the operation. Immediately after the operation he fell asleep, and we left him soon after midnight." "Jan. 5th. 5, A. M. Saw him again. Still asleep. He remained comfortable during the day, waking now and then, and drinking milk and water. * * * 6, P. M. Breathing rapid and very difficult. Removed the tube, and with great exertion, he blew out through the opening about a drachm of slightly bloody membrane. Introduced a double tube. 9, P. M. Asleep and breathing very quietly; has rejected shreds of lymph by the mouth. * * * * If the tube be closed, no air passes the glottis." Brevity compells an omission of most of the further details. "On the 10th, on closing the tube, his effort to breathe forces air through the glottis with difficulty, and with a rattling sound." "Jan. 12th. Closure of the tube causes excessive dyspnœa and cough, which is accompanied with a flapping sound." "Jan. 19th. Can bieathe moderately well with the tube closed. * * * Purulent discharge from the wound, rather profuse." "Jan. 21st. 1, P. M. Asleep. Corked the tube. He was much alarmed, and began to cough and cry *audibly*, for the first time. Breathing very hoarse, but without apparent difficulty. Removed the tube, and left the wound open." "Jan. 22d. No air passes the wound. Scab forming over it." "Jan. 28th. Consider him well. Nothing peculiar observable, except that his voice is hoarse and feeble." "In the case above related, no medicine was used from the time of the operation. The pleasant result of it is undoubtedly to be attributed to the fact, that the operation was performed as a means of treatment, and not as a last resort. I have seen a sufficient number of fatal cases of croup to feel certain that a persistence in medication would have been useless. The state of the patient's skin, his inability to vomit, the absence of cough and the intermittent pulse, made me doubt whether his nervous system were not too much depressed from the first, to warrant much probability of success. In addition to this there was some difficulty in keeping up respiration, which came on some five minutes after the tracheal tube was inserted. This may in part have been caused by the etherisation. Without ether, it would not have been possible to operate. The free use of cold water upon the abdomen and chest by sprinkling, had more effect than anything else in exciting the respiratory act, although the 'ready method' was resorted to."

The above admirably conducted case, is, particularly when the age of the child is taken into account, the best instance the writer has been able to find upon record, in either European or American practice, of the occasional great efficacy of tracheotomy in the disease under consideration. As such he has felt that he is not trespassing upon your time in giving Dr. Buckingham's nearly unabridged account of it.

In view of the foregoing considerations, the question of "tracheotomy in membranous croup," gives rise to the following interrogatories:

1. Under what circumstances, if any, should it be made, and by what complications is it absolutely prohibited?

2. What subsequent measures may be regarded as accessory or necessary?

3. Is the operation in itself or in its consequences hazardous to life?

In order to reply fully to the first inquiry, it seems important to recapitulate, and to weigh, several of the points that have been assumed or referred to in this paper.

Adopting the hypothesis of the "croupous crasis," if not absolutely, at least partially, must we therefore with M. Piorry and Jules Guerin, question the propriety of employing tracheotomy for the relief of a local manifestation of a general disorder. Their mode of reasoning might with almost equal propriety be urged against endeavors to divert gout or rheumatism from the stomach or endocardium. Is the fact that the cavity of the trachea is almost invariably patent to a considerable degree, a valid objection? It will be remembered that a membrane, even if small, may, if one extremity become free, prevent egress or ingress of air by valvular action. It will also be borne in mind, that false membrane, acrid or inspissated mucus, or even an inflamed or turgid condition of the lining of the larynx unattended by secretion or exudation, unquestionably may give rise to long and repeated spasmodic occlusion of the glottis, a condition full of danger, which tracheotomy would under harmless. It next seems necessary to glance at the mortality of the affection. This, under various forms and combinations of treatment, Dr. Green's method possibly excepted, may be set down at two-thirds of all attacked. What the natural termination would be, can only be a matter of inference; it is probable, however, that it would be fatal in nearly every instance, yet Dr. Ware tells us, that one of his patients who was extremely ill, recovered spontaneously, he being so ungovernable as to successfully resist all attempts at medication; and your reporter has also a case of unassisted recovery to cite: Some years ago he visited a child, two years of age, who had been ill several days; nothing whatever had been done, recovery was deemed impossible; the infant was apparently moribund; no prescription was made. The next day he was better, and inspection revealed thick false membrane upon the tonsils. The child recovered, but for three weeks was unable to cry audibly, and his voice was weak and hoarse for two months. Your reporter does not know that it is necessary to explain his inaction, but he feels it due to himself to say, that while he made frequent visits, a "masterly inactivity" seemed to him his wisest and safest course in that particular and solitary instance.

Let us now look at the results of tracheotomy, and let us *assume*, that three-fourths of those upon whom the operation is made, perish, although the statistics of Valleix make the proportion consideraly less. We must remember that it has usually been employed as a last resource, when all other measures have failed, many of which, although proper in themselves, must to a certain extent, prejudice the success of the operation. Tracheotomy should not be *contrasted* with the other means of treating croup, as being one mode in distinction from others, but it should be looked upon as a measure not to be neglected when all other rational treatment is evidently powerless; and in this aspect, to use the strong language of Mr. Henry Smith, of London, a success of but one in ten or even one in twenty, justifies the procedure. The proper moment is not so much a matter of time as of symptoms; it may be within twenty-four hours of the seizure, or many days from the commencement of the attack. If, after general treatment, and especially topical treatment, has been fully and faithfully tried, the symptoms of suffocation are not relieved or are becoming more threatening, and especially if with persistent dyspnœa, there are manifestations of cyanosis, the operation should not be delayed. If, however, what may be termed the proper period has passed, may the trachea still be opened with any hope of permanent advantage? To this it may be replied, that if the symptoms are still chiefly referable to the upper portion of the air passages, although conjoined with great exhaustion, it is advised by those who have practised it most, although from the previous failure of vital force, the patient may succumb to the shock of the operation. The age of the patient is an important consideration. The older the person, *cæteris paribus*, the greater the propability of a successful issue. Children under two years old "invariably die," says Trousseau, and such is the universal testimony. It is probable that the severity of the operation is too great for their tender age. It is possible that with them laryngotomy might, with advantage, be substituted for tracheotomy, as comparatively it is easily made, and does not involve much time or loss of blood in its performance. It is, indeed, a question whether it might not in every instance take the place of tracheotomy. It would relieve spasm of the glottis quite as effectually, and the success of tracheotomy does not depend upon the position of the external orifice, as being below the membranous formation. Thus, "Dr. Craig has published a very interesting case of successful tracheotomy, in which the membranes reached an inch below the lowest point of the incision," and "M. Soloy has related a successful case of laryngotomy where the membrane also extended below the incision." (Bemiss' Essay loc. cit.)

The complications prohibiting tracheotomy are rather those connected with the nervous and circulatory than with the pulmonary apparatus. These may be laid down as follows: delirium, or stupor, excessive prostration, feeble and very frequent pulse, coldness and palor or great lividity of the surface. With reference to bronchitis and pneumonia there is a diversity of opinion. It is held by M. M. Rilliet and Barthez, as also by M. Guersent, that the extension of the membrane to the smaller bronchia, or pneumonia of a single lung, are not to be regarded as insuperable objections, although they certainly diminish the chances of recovery. All persons who have seen much of membranous croup, will admit the difficulty, and often the impossibility, of determining, by physical exploration, the exact condition of the lungs. The youth and restlessness of the patient, the hurried and imperfect respiration, and the loud laryngo-tracheal breathing, the latter especially by masking or overpowering all other sounds, render the task always difficult and often abortive. Flatness on percussion, may be due to collapse of lung rather than to solidification from congestion or fibrinous exudation, and sibilant and mucous râles, which may during life have been heard all over the chest, are often proved, on post mortem examination, not to have proceeded from the smaller air tubes. As these are assertions which will doubtless be disputed, and especially by those least familiar with ausculta- tion, but who arrogate for themselves thorough acquaintance with it, the writer feels called upon to substantiate his position, from the article of Dr. J. F. Meigs, (p. 113, 3d Ed.)

"The first case is one mentioned by MM M. De La Berge and Mouneret, in which they could not believe that the bronchia contained false membranes, as the vesicular murmur was extremely pure, and was heard everywhere; and yet during the operation a false membrane was drawn out which represented the trachea and the division of the principal bronchia. The child died in 15 hours. Dr. Wm. Pepper, of this city, reports two fatal cases, in one of which 'distinct vesicular murmur could be heard throughout the lungs, marked only occasionally by sibilant and sonorous rattles,' a few hours before tracheotomy was performed. The child died twenty hours after the operation, and the exudation was found to implicate the larynx, trachea, the large bronchia and even some of the smaller ramifications. In the other case, the state of the respiration was carefully examined the day before death, and not the least respiratory murmur could be heard over any part of the chest, and yet, in this instance, the exudation was confined strictly to the larynx—not a vestige of false membrane was to be found either in the trachea or bronchia."

These cases illustrate the writer's assertions only so far that they demonstrate the falsity of deductions drawn from the presence or absence of certain natural or morbid sounds. But to return to the subject of pulmonary complication. There seems to be one valid argument against operating when either bronchitis or pneumonia are supposed to exist, viz., the success that M. Trousseau claims to have accomplished since the adoption of the woolen cravat, which is used exclusively to prevent the supervention of pulmonary disease.

In reply to the second inquiry, respecting accessory treatment, may be mentioned—a well ventilated apartment, maintained at an equable temperature ranging from 70° to 75°, the atmosphere being kept moist by the evaporation of warm water; the adaptation of a light coarse woolen muffler about the throat, and enforced alimentation if food is not taken readily. If from any cause respiration is suddenly suspended, whether immediately succeeding the operation or some time subsequently, the lungs should be carefully inflated through the artificial opening, or if suspension is produced by clogging of the trachea or larger bronchia, an attempt should be made to remove the obstacle by inserting a large flexible tube, open at both ends, into the trachea, and drawing upon it quickly and forcibly with the mouth. Roux saved a patient by this expedient, removing a clot of blood with a female catheter. In several instances, in the United States, the patient has been kept under the influence of chloroform or ether, and although most of those to whom the anæsthetic was given, died, in no case has the fatal event been attributed to the inhalation. It certainly must greatly facilitate the operation, as well as diminish the shock. It will be remembered that in the admirable case reported by Dr. Buckingham, he states that he could not have got on without it. At present there appears to be no objection to its use, and it is hoped that while it robs this formidable operation of all its terrors, it may also do away with much of its danger, for, in answer to our third interrogatory, there can be but this response—it is *in it-*

*self* hazardous to life—hazardous from shock, from exhausting hæmorrhage, and from inspiration of blood. M. Guersent frankly avows these, and while M. Trousseau speaks of it lightly, his own account, and his own directions contradict him. Surgeons, everywhere, almost without exception, declare it to be a serious and formidable and bloody operation, and such it undoubtedly is, notwithstanding several notable exceptions. It is probably also more hazardous to life in its consequences, than equally severe operations in other portions of the body. This is sufficiently indicated by the unusual precautions employed by its chief advocate, M. Trousseau; but admitting the whole hazard, both of the operation and its consequences, the question recurs, as to the comparative risk of the operation or its neglect. To this your reporter would respectfully submit, that a full and he trusts impartial investigation of the subject impels him to the conclusion, that, Tracheotomy, from its intrinsic gravity, never to be lightly or precipitately undertaken, affords a possibility of recovery, when hope from other resources cannot reasonably be entertained, and that moment having arrived, it should not be neglected or delayed.

